# Mental Health Outcomes of a National Cohort of Adults Born with Very Low Birthweight

**DOI:** 10.3390/jcm13247591

**Published:** 2024-12-13

**Authors:** Georgina L. Moody, John Horwood, Sarah L. Harris, Brian A. Darlow, Lianne J. Woodward

**Affiliations:** 1 Department of Psychological Medicine, University of Otago Christchurch, Christchurch 8140, New Zealand; georgina.moody@cdhb.health.nz (G.L.M.); john.horwood@otago.ac.nz (J.H.); 2 Specialist Mental Health Services, Te Whatu Ora Waitaha, Christchurch 8011, New Zealand; sarah.harris@otago.ac.nz; 3 Department of Paediatrics, University of Otago Christchurch, Christchurch 8140, New Zealand; 4 Canterbury Child Development Research Group, School of Health Sciences, University of Canterbury, Christchurch 8140, New Zealand; lianne.woodward@canterbury.ac.nz

**Keywords:** population-based cohort study, very low birthweight infants, preterm young adults, follow-up, mental health, anxiety disorders

## Abstract

**Background:** Children born with a very low birthweight (VLBW; <1500 g) and/or very preterm (VPT; <32 weeks) are at increased risk of mental health problems, but adult data are inconsistent. **Objectives:** We examined the prevalence of a range of mental health disorders in a national cohort of adults born with a VLBW, as well as associations between gestational age and mental health outcomes. **Methods:** All infants born with a VLBW in New Zealand in 1986 were followed prospectively from birth. The 12-month prevalence of mental health outcomes, in addition to substance use and violent/property offending were assessed at a median age of 28 years in this cohort relative to 100 full-term (FT) controls. Outcomes were assessed using structured interview methods. **Results:** There was a modest increase in the overall rate of mental health problems in VLBW adults compared to controls (ARR 1.33 [95% CI 0.83, 2.12]), reflecting slightly higher rates of anxiety disorders, notably agoraphobia (ARR 2.98 [0.64, 13.85]), social phobia (ARR 1.61 [0.71, 3.65]), and suicidal ideation/attempt (ARR 1.66 [0.45, 6.08]), but not depression (ARR 1.02 [0.57, 1.81]). There were no clear differences in substance use/offending outcomes. VLBW individuals born extremely preterm (<28 weeks) were most vulnerable to later mental health problems relative to controls (overall rate of mental health problems ARR 1.54 [0.86, 2.73]). Effect sizes for any anxiety disorder were also higher for VLBW females than VLBW males compared to same-sex controls. **Conclusions:** This population-based longitudinal cohort study showed that adults born preterm with a VLBW reported more mental health problems than FT controls; however, this difference was small. Pooled analyses involving larger sample sizes are needed, but findings suggest only modest individual and public health impacts of preterm birth on adult mental health functioning.

## 1. Introduction

Understanding the longer-term outcomes of infants born with very low birthweight (VLBW; <1500 g) and/or very preterm (VPT; gestational age < 32 weeks) is important for counselling families and anticipating future health needs. It is also important from a public health perspective, given that worldwide around two million infants are born with a VLBW/VPT [1], with a high proportion (around 40%) of these infants experiencing later childhood neurodevelopmental difficulties [2]. Children and adolescents born with a VLBW/VPT are at greater risk of mental health disorders and symptoms, with 25–37% having at least one disorder [3,4,5,6,7]. The most common of these are attention deficit hyperactivity disorder (ADHD), autism spectrum disorder (ASD), and anxiety/mood disorders [3,4,5,6]. Conversely, VLBW/VPT adolescents appear less susceptible to problematic alcohol and substance use and criminality [4,5,8,9].

Whilst mounting evidence suggests that many of the neurodevelopmental difficulties seen in VLBW/VPT children persist into adulthood [10,11,12,13], the profile for other adult mental health outcomes is less certain. Beyond the psychological impact, mental health challenges can negatively affect physical health and socioeconomic success. Understanding the impact of premature birth on mental health across the lifespan could inform healthcare policies that recommend screening and support to those groups most at risk. Existing birth cohort studies examining adult mental health outcomes have produced mixed results [14,15,16,17,18,19]. They have also often been limited by small sample sizes (n < 150 VLBW/VPT participants in most studies) and variability in measurement quality, sample representativeness, and the extent of control for confounding variables [15,16]. Larger registry studies show a modest increase in psychiatric risk [8,20,21], hospitalisation [22], and psychotropic medication prescription [23,24] amongst VLBW/VPT adults. Although these studies have greater statistical power, their reliance on official record data likely underestimates the extent of mild–moderate mental health disorders, and inter-clinician reliability is not high for psychiatric diagnosis where structured interview methods are not applied. These studies also do not typically differentiate between adolescent and adult outcomes. There is a need for larger population-based cohort studies, which take account of the cultural context (Māori are 18% of the overall New Zealand population) and which examine a range of adult mental health outcomes using psychometrically valid, structured interview methods. The aims of this study were the following:

1. To compare the 12-month prevalence of a range of mental health disorders, in addition to substance use and violent/property offending behaviour in a national cohort of adults born with a VLBW relative to a control sample of same-age full-term (FT) adults;
2. To examine whether the risk of adverse mental health outcomes differed for those born extremely preterm (EPT; <28 weeks’ gestation) and/or with an extremely low birthweight (ELBW; <1000 g).

## 2. Methods

### 2.1. Case Control Selection

The case sample comprised a national cohort of individuals born with a VLBW and admitted to neonatal intensive care units (NICU) throughout New Zealand during 1986 and then studied prospectively up to a median age of 28 years. Of the 413 infants born with a VLBW and admitted to a NICU, 338 (82%) survived to discharge. At age 28, 250 (77% of 323) survivors were assessed. A comparison sample of same-age full-term (FT) born adults were initially recruited at an age of 22 years and further increased to n = 100 at the 28 year assessment via a process of peer-nomination and random sampling from electoral rolls, with efforts to balance for sex, ethnicity, and regional location [25]. Figure S1 describes our cohort recruitment and retention.

### 2.2. Exposure

The primary exposure was case status (VLBW vs. FT control) in comparisons of outcomes for the full study sample. The sample was also stratified by biological sex. In further analyses, the VLBW group was subdivided by gestation (<28/≥28 weeks) and birthweight (<1000/1000–1499 g).

### 2.3. Outcomes

At a median age of 28 years (range: 26–30), 250 VLBW young adults and 100 FT controls completed a comprehensive face-to-face interview assessing aspects of their physical and mental health, psychosocial adjustment, and life circumstances. Social and demographic characteristics were collected for all participants using self-report and/or parent/significant other reports.

Participant mental health over the preceding 12 months was assessed using components of the Composite International Diagnostic Interview (CIDI) [26] schedule and custom written survey items previously validated in the New Zealand-based Christchurch Health and Development Study [27]. CIDI subscales provided standardised measures of a range of mental health disorders, including major depression, generalised anxiety disorder, panic disorder, agoraphobia, social phobia, and specific phobia. Custom written items assessed suicidal ideation/attempt/s, offending behaviour, tobacco use, and alcohol and substance use/misuse, including the prevalence of weekly alcohol binge drinking, daily cannabis use, and any other illicit drug use in the last 12 months [27].

Participants were also questioned about any other psychiatric diagnoses or mental health problems and associated hospitalisations in the past 12 months. Medications and medical/health professional contacts (e.g., counsellor and psychologist) were recorded and independently reviewed by a psychiatric registrar (GM) to cross-check CIDI data and potentially identify additional psychiatric diagnoses. Individuals who were receiving medication for anxiety and/or depression but did not meet full CIDI diagnostic criteria were classified as exhibiting subthreshold symptoms of anxiety and depression.

### 2.4. Statistical Analysis

Differences in perinatal and demographic factors between VLBW and control groups and between VLBW assessed/not assessed cases were summarised by the mean or risk difference and 95% CI. Comparison of outcomes between the VLBW (or sub-groups of VLBW) cohort and controls was conducted using the rate ratio (RR) and 95% CI as the measure of effect size (ES). The control sample was the reference group for all comparisons. Observed ESs were adjusted for potential differences in baseline characteristics (assessment age, sex, ethnicity, breastfeeding, maternal age at childbirth, parental education, and childhood family socioeconomic status) using logistic regression for dichotomous outcomes and Poisson regression for count outcomes, with adjusted rate ratios (ARRs) for dichotomous outcomes calculated using the methods described by Norton et al. [28]. RRs were preferred to the more traditional odds ratio (OR) as the reported ES measure for dichotomous outcomes since not all outcomes were of low prevalence, and for higher prevalence outcomes, the OR provides a biased estimate of the true RR [28]. Where relevant, tests for possible statistical interactions were conducted by testing the equality of ESs between sexes or between sub-groups of the VLBW cohort. Sensitivity analyses were conducted to examine whether the exclusion of those VLBW participants with a prior history of moderate/severe neurosensory disability at age 7–8 impacted observed between-group differences in adult outcomes. In addition, E-value methods were used to consider the possibility of unmeasured confounding [29]. Statistical analyses were conducted using SAS v9.4 and Stata v17.

### 2.5. Missing Data

Missing data comprised less than 1% of all outcomes (all in the VLBW sample). Comparisons of those VLBW survivors assessed at age 28 with those not assessed (Table S1) provided some evidence of selection bias. Specifically, survivors seen at age 28 had lower mean birthweight and were more likely to be female. To address issues of missing data and potential selection bias in the VLBW sample, the adjusted ES analyses also included an inverse probability weighting adjustment for the VLBW group. Specifically, for each outcome, a logistic regression model was first fitted to predict the probability of inclusion in the assessment sample from the measures in Table S1. The inverse of this probability was then used as the weight for each individual in the adjusted regression models.

### 2.6. Ethics Approval

Written informed consent was obtained from all study participants, and all procedures were approved by the Upper South B Regional Ethics Committee, Wellington (Ethics Ref: URB/12/05/015).

## 3. Results

There were 250 (107 male) VLBW participants, with a mean age at assessment of 28.5 years, and 100 (37 male) term-born controls, with a mean age at assessment of 28.2 years. Table 1 describes the neonatal and social background characteristics of the sample. Table 2 shows the mental health, substance use, and offending outcomes of VLBW adults and their FT peers at a median age of 28 years. Over the preceding 12 months, VLBW adults reported higher rates of a range of DSM-IV anxiety disorders and twice the rate of suicidal ideation, although these differences were modest (RRs range: 1.1–2.6). After adjustment for the effects of age, sex, ethnicity, breastfeeding, maternal age at childbirth, parental education, and family socioeconomic status, in addition to potential selection bias in the VLBW sample, VLBW young adults had an overall mean rate of mental health problems that was 1.33 [95% CI 0.83, 2.12] times that of FT controls. The largest effect sizes were observed for agoraphobia (ARR 2.98 [95% CI 0.64, 13.85]), social phobia (ARR 1.61 [0.71, 3.65]), and suicidal ideation/attempt (ARR 1.66 [0.45, 6.08]). No between-group difference was found for major depressive disorder before or after covariate adjustment.

With respect to substance use and offending outcomes, while VLBW adults had slightly elevated rates of daily cigarette use and weekly binge drinking (RRs 1.4), these differences largely disappeared after controlling for covariates. Both groups had similar rates of cannabis and other illicit drug use and adult offending.

Given the observation in Table 2 that VLBW adults reported small but consistent elevations in rates of anxiety, the possibility of differences in subthreshold difficulties was explored (Table S2). The inclusion of those with subthreshold symptoms produced a stronger association for generalised anxiety (ARR 1.71 [0.65, 4.49]) and was also slightly increased for social phobia (1.78 [0.96, 3.30]) and panic (1.60 [0.65, 3.93]) but not major depression 1.01 [0.65, 1.56].

When sex differences were examined (Table S3), the overall rate of mental health problems amongst VLBW adults compared to FT controls of the same sex was similar for males and females (ARRs 1.35–1.36). However, there was some evidence of between-sex variation in RR by type of disorder. For anxiety disorders, effect sizes for VLBW females (relative to same-sex controls) were consistently stronger than for VLBW males, most notably for agoraphobia (ARR females 6.01 [0.81, 44.62]; males 0.07 [0.00, 1.56]). In contrast, VLBW males were at higher relative risk of depression (ARR males 1.92 [0.54, 6.82]; females 0.78 [0.40, 1.54]). Similarly, while VLBW males exhibited modestly elevated rates of tobacco and other illicit substance use compared to their FT same-sex peers (ARRs 1.50, 1.99), the opposite was true for females (ARRs 0.97, 0.52). However, tests of sex-by-group-status interactions showed that none of these sex differences were statistically significant.

In further analysis, the VLBW group was stratified by gestational age (GA) into two subgroups, <28 weeks GA (EPT) and ≥28 weeks GA, to examine the extent to which outcome risks relative to FT controls increased with the degree of prematurity (Table 3). There was a clear pattern in which those born EPT exhibited higher RRs on most mental health outcomes compared to those born at higher gestational ages. After covariate/selection bias adjustment, the overall rate of mental health problems in EPT vs. FT controls was ARR 1.54 [0.86, 2.73] compared to ARR 1.25 [0.76, 2.03] for those VLBW adults born ≥28 weeks GA.

Substance use and offending outcomes showed no clear pattern of association with the extent of prematurity. The overall rate of these outcomes relative to FT controls was similar in both the <28 week and ≥28 week GA VLBW groups (ARRs 0.98 [0.65, 1.49] and 0.92 [0.64, 1.33], respectively).

A parallel analysis stratifying the VLBW group by birthweight showed no consistent evidence of variation in outcome RRs between those born ELBW (<1000 g) and other VLBW adults (1000–1499 g) (Table S4). The overall rate of mental health problems relative to FT controls was similar for both groups (ARR ELBW 1.38 [0.75, 2.51]; other VLBW 1.31 [0.81, 2.12]), as was the overall rate of substance use/offending outcomes (ARR ELBW 0.92 [0.57, 1.48]; other VLBW 0.94 [0.65, 1.36]).

Sensitivity analyses excluding those adults born with a VLBW subject to moderate to severe neurosensory disabilities at age 7–8 years did not substantively affect the findings (Table S5). Finally, consideration of unmeasured confounder bias [29] applied to the overall rate of mental health problems in Table 2 (ARR 1.33) gives an E-value of 2.0, and for the individual outcome with the strongest ES (agoraphobia, ARR 2.98), an E-value of 5.2 is obtained. This suggests that for these associations to be explained away by an unmeasured confounder would require that the confounder had moderate to strong associations (respectively) with both group status and the outcome, independent of other confounders. However, for both outcomes, the E-value for the lower limit of the CI is 1, suggesting that the E-value estimates are relatively imprecise.

## 4. Discussion

This population-based longitudinal cohort study showed that 28-year-old adults who were born preterm with a VLBW reported more mental health problems than FT controls; however, this difference was small. When subthreshold symptoms were included, RRs remained similar suggesting that the later clinical risk of mental health disorder is at most modest, with the majority of VLBW adults having similar psychological wellbeing to their term-born adult peers by their third decade of life. When relations between gestational age and later mental health outcomes were examined, the extremely preterm group appeared at greater risk, with a 54% higher mean rate of mental health disorder than their same age FT control peers.

Whilst preterm birth confers an increased risk of psychiatric symptoms and disorder in childhood, the extent to which these risks persist into adulthood has been less clear. During childhood and adolescence, VLBW/VPT individuals are more likely to be shy, unassertive, withdrawn, anxious/depressed, and experience social difficulties than their peers [5,6,7,9,30,31,32,33,34]. They are also less likely to engage in risk-taking and delinquent behaviours [9]. Existing research also supports an increased rate of social difficulties and withdrawal across the life-course for VLBW/VPT individuals which is most apparent in ELBW/EPT survivors [9,18,25,32,33,34]. As VLBW/VPT individuals transition into adulthood, they stay longer in the parental home, tend to have fewer friends and sexual partners, and are slower to form intimate romantic relationships [8,9,18,25,35,36]. A meta-analysis [37] of self-report questionnaire data (n = 6 studies) found that VPT-born young adults showed modest increases in internalising and avoidant personality problem scores and lower externalising problems scores than FT controls. A recent systematic review [18] of cohort (n = 11) and registry studies (n = 2) reported higher lifetime rates of psychotropic medication use in VLBW/VPT adults, but inconsistent results with respect to risks for mental health disorders and symptoms.

A limitation of the existing research is the small sample size of the cohorts, with Robinson’s review [18] only including two birth cohorts with more than 200 VLBW/VPT participants. A recent registry study [20] found modest associations between gestational age at birth and psychiatric disorder risk. In contrast, a prospective follow-up study [16] of a regional cohort of 174 ELBW/EPT young adults at age 25 relative to 139 FT controls found similar rates of ADHD, anxiety, and mood and substance use disorders in both groups based on self-report and structured interview measures. These findings suggest similar or only modest increases in mental health issues in adults born with a VLBW compared to control groups, consistent with our findings. However, to confirm this and explore specific disorders, there is a need for larger population-based cohort studies of adult outcomes and/or for data pooling across existing birth cohort studies to increase statistical power.

Although our examination of specific mental disorders was relatively underpowered for lower prevalence outcomes, the findings do suggest a pattern towards a modest increase in mental health disorders/symptomatology in VLBW adults relative to FT controls, reflecting small increases in anxiety disorders, most notably agoraphobia, social phobia, and suicidal ideation. There were also weak indications that the rates of anxiety disorders relative to same-sex FT peers may be greater for VLBW females than males. These results are generally consistent with data from registry studies [20,21] which report a small increase in the risk of stress-related and somatoform disorders. They are also in line with a recent IPD meta-analysis [14], which found a small but significant increase in the odds of anxiety disorder (OR 1.44) in VLBW/VPT individuals over 18 years relative to controls.

With respect to major depressive disorder, we found similar rates across both the VLBW and FT groups. However, when risks were examined in relation to the extent of prematurity, there was some evidence that VLBW adults born EPT may be at increased risk of depressive disorder/symptoms (21% versus 13% of those born ≥28 weeks GA and 15% of FT controls). A consensus has yet to be reached as to whether VLBW/VPT birth confers an increased risk of depression [12]. Existing cohort studies report mixed results [12], although meta-analyses [14,19] suggest slightly elevated levels of depression when data across studies are pooled. Loret de Mola et al. [19] found a significantly higher rate of depression in adults of low birthweight (OR = 1.39) but did not observe a linear relationship between birthweight and depression. We have previously reported that exposure to antenatal corticosteroids in this VLBW cohort was associated with an increased risk of depression (ARR 2.03, 95% CI 0.99, 9.88) compared with non-exposure, and that there was a slightly stronger association with EPT birth in adults [38]. Further clarification of this issue is clearly needed.

Although some studies have found lower levels of risk-taking and criminality amongst VLBW adolescents and young adults [8,9], we found a generally similar pattern of substance use and offending across VLBW and control FT adults at age 28 when sociodemographic factors were taken into account. However, when sex differences were analysed, there was weak evidence of a possible interaction whereby VLBW males showed a modest elevation in the rate of tobacco and illicit drug use relative to same-sex FT peers, whilst the opposite was true for VLBW females.

Observed associations between VLBW/VPT and mental health vulnerability likely reflect the complex interplay of biological and psychosocial mechanisms. Specifically, VPT birth has been shown to impact neonatal brain structural development and neural connectivity in key regions involved in emotional processing and social functioning [38,39], which in turn may potentially contribute to later emotional and behavioural difficulties [40,41,42]. Exposure to stress and pain in the NICU may also play a role given that abnormal activation of the hypothalamic–pituitary axis (HPA) in early life is a well-recognised risk factor for both internalising and externalising behaviours [43,44,45].

With respect to potential psychosocial and postnatal environmental factors, preterm birth is associated with elevated levels of parental anxiety, stress, and parent–infant attachment difficulties that may increase a child’s vulnerability to anxiety [46,47,48]. For example, disrupted attachment has been implicated in the development of a range of mental health conditions, including depression, anxiety, and personality disorder/dysfunction. Similarly, the Canterbury preterm study [48] showed that maternal mental health was an important independent predictor of early-onset child anxiety at age 9 years. Further research examining these potential processes will be informative.

Strengths of our study included a population sample with a multi-ethnic make-up similar to the New Zealand population, high sample retention, multi-source reporting, and use of validated structured-interview methods. In addition, analyses have taken into account important potential confounders including neurosensory disability and social background.

There are several limitations of this study. First, despite our comparatively large population-based sample, our study had limited power to detect small between-group differences or to conduct subgroup analysis. This is reflected in the relative imprecision of RR estimates. Secondly, missing data (<1%) and differential loss to follow-up (73 unassessed cohort members) were a potential source of bias. However, the present analysis utilised data weighting methods to take these factors into account. Furthermore, comparison of the ES estimates from these analyses with the equivalent estimates from analyses that did not apply the weighting correction showed negligible differences between the two sets of ARRs. This suggests that in this instance selection bias was not a major threat to study validity. Thirdly, we did not directly question participants about symptoms of ADHD and ASD at age 28. Despite efforts to capture this information through questioning about other previous or current mental health issues, medications, and hospitalisations, these data had low reliability and were not included. We did, however, assess ADHD at 22–23 years when a face-to-face interview incorporated the Barkley adult self-report scales of attention deficit/hyperactivity disorder [19]. Our results contributed to an individual patient data (IPD) meta-analysis involving eight adult preterm studies (1385 preterm; 1513 term-born) which reported similar levels of self-reported ADHD symptoms in preterm individuals and term-born controls [15]. Fourthly, we were unable to consider the role of genetic susceptibility. A first-degree family history of mental health disorder is a strong risk factor for mental health problems and is also correlated with other risk factors for preterm birth such as socioeconomic disadvantage. Although existing research generally suggests that genetic predisposition does not explain observed between-group differences in mental psychopathology in this high-risk population [7,8,22], it should be considered a potential confounder in future analyses. Finally, we note that cross-checking of respondent interview data revealed some inconsistencies in self-reports and a lack of specificity around diagnoses. For example, two participants responded ‘no’ to experiencing ‘any other previous mental or physical health problem’, but one later reported a psychiatric hospitalisation for ‘severe behaviour and paranoia’ and the other later reported being on medication for a psychiatric indication. This observation highlights the importance of cross-checking self-reports and health records where possible, along with inclusion of other significant data, as was the case in our study, to optimise data accuracy.

## 5. Conclusions

Our findings indicate that despite modest elevations in some anxiety disorders and suicidal ideation, the majority of adults born VPT/VLBW do not experience mental health disorder. Further research and pooled analyses employing larger sample sizes are important to better understand the long-term implications of VPT/VLBW birth for adult mental health and to inform clinical practice and public health policy, particularly with regard to lower frequency outcomes. Although, with access to intensive care, the survival of those born with a VLBW is high, subtle morbidity may persist into adulthood across a range of domains. Recognising this is important to enable targeted public health investment to ensure premature birth does not confer lifelong disadvantage. Overall, it is encouraging to note that most infants born with a VLBW appeared to be experiencing reasonable mental health in adulthood that was comparable with their FT peers.

## Figures and Tables

**Table 1:** Demographic and perinatal characteristics of VLBW adults and controls.

Measure	VLBW (n = 250)	Controls (n = 100)	Difference (95% CI)
Age at assessment, mean (SD), years	28.5 (1.1)	28.2 (0.9)	0.29 (0.04, 0.53)
Male, %	42.8	37.0	5.8 (−5.5, 17.1)
Māori/Pacific Island ethnicity, %	30.8	24.0	6.8 (−3.3, 16.9)
Birthweight, mean (SD), g	1134 (236)	3377 (584)	−2243.3 (−2332.4, −2154.1)
<1000 g, %	27.2	-	
Gestation, mean (SD) weeks	29.2 (2.5)	-	
<28 weeks gestation, %	26.0	-	
Small for gestational age (<10th centile), %	30.0	-	
Antenatal corticosteroids, %	56.4	-	
Respiratory distress syndrome, %	56.4	-	
Bronchopulmonary dysplasia ^a^, %	20.4	-	
Retinopathy of prematurity, %	21.6	-	
Breastfed, %	76.4	88.0	−11.6 (−19.9, −3.3)
Maternal age at birth, mean (SD), years	25.9 (5.2)	27.1 (4.5)	−1.20 (−2.36, −0.04)
Parent with tertiary education, %	52.0	63.0	−11.0 (−27.3, 0.3)
Family socioeconomic status ^b^, mean (SD)	3.3 (1.5)	3.1 (1.3)	0.27 (−0.08, 0.62)

VLBW: very low birthweight, SD: standard deviation. ^a^ Oxygen requirement at 36 weeks post-menstrual age. ^b^ Scored using a 6-level classification ranging from 1 = professional to 6 = unskilled occupational status.

**Table 2:** Mental health, substance use, and offending outcomes in VLBW adults and controls.

Measure	VLBW (n = 248–250 ^a^)	Controls (n = 100)	RR (95% CI)	
			Unadjusted	Adjusted ^b^
**Mental health**				
12-month prevalence, %				
Generalised anxiety disorder	4.8 (n = 248 ^a^)	4.0	1.21 (0.40, 3.66)	0.83 (0.26, 2.63)
Panic disorder	4.4	4.0	1.10 (0.36, 3.37)	1.40 (0.43, 4.54)
Agoraphobia	5.2	2.0	2.60 (0.60, 11.31)	2.98 (0.64, 13.85)
Social Phobia	13.7 (n = 249 ^a^)	7.0	1.95 (0.89, 4.25)	1.61 (0.71, 3.65)
Specific phobia	12.4	11.0	1.13 (0.59, 2.15)	1.36 (0.71, 2.63)
Any anxiety disorder ^c^	26.4	21.0	1.26 (0.82, 1.94)	1.27 (0.81, 1.99)
Major depression	15.3 (n = 249 ^a^)	15.0	1.02 (0.59, 1.76)	1.02 (0.57, 1.81)
Suicidal ideation ± attempt	6.4	3.0	2.13 (0.64, 7.16)	1.66 (0.45, 6.08)
Number of mental health problems ^d^ (past 12 months), mean (SD)	0.63 (1.05) (n = 248 ^a^)	0.46 (0.93)	1.36 (0.98, 1.89)	1.33 (0.83, 2.12)
**Substance use and offending**				
Substance use, % (past 12 months)				
Daily cigarette smoker	30.0	21.0	1.43 (0.93, 2.18)	1.12 (0.75, 1.66)
Weekly binge drinking ^e^	15.6	11.0	1.42 (0.76, 2.66)	1.19 (0.63, 2.23)
Daily cannabis use	7.6	8.0	0.95 (0.43, 2.10)	0.68 (0.30, 1.53)
Other illicit drug use ^f^	11.2	11.0	1.02 (0.53, 1.97)	0.86 (0.43, 1.72)
Offending, %				
Property or violent offences (since age 18)	22.4	23.0	0.97 (0.64, 1.49)	0.78 (0.51, 1.18)
Number of substance use/offending problems, mean (SD)	0.87 (1.20)	0.74 (1.14)	1.17 (0.90, 1.53)	0.94 (0.66, 1.33)

VLBW: very low birthweight, RR: rate ratio, CI: confidence interval, and SD: standard deviation. ^a^ n < 250 for some measures where participants or parent proxies only completed part of the interview. ^b^ Adjusted for sex, ethnicity, breastfeeding, maternal age at childbirth, parental education, childhood family socioeconomic status, and potential selection bias in VLBW. ^c^ Generalised anxiety disorder, panic disorder, agoraphobia, social phobia, and/or specific phobia assessed using standardised diagnostic criteria. ^d^ Suicidality, depression, and/or anxiety disorders listed above. ^e^ Defined as consuming ≥6 standard drinks on >50 occasions in the last year. ^f^ Use of any illicit substance other than cannabis and/or abuse of prescription medications for recreation.

**Table 3:** Mental health, substance use, and offending outcomes stratified by gestational age ^a^.

Measure	VLBW < 28 Weeks (n = 65)	VLBW ≥ 28 Weeks (n = 183–185 ^b^)	Controls ≥ 37 Weeks (n = 100)	VLBW < 28 Weeks vs. Controls		VLBW ≥ 28 Weeks vs. Controls	
				RR (95%CI)	ARR (95%CI) ^c^	RR (95%CI)	ARR (95%CI) ^c^
**Mental Health**							
12-month prevalence, %							
Generalised anxiety disorder	6.2	4.4 (n = 183 ^b^)	4.0	1.54 (0.40, 5.94)	1.25 (0.30, 5.20)	1.09 (0.34, 3.54)	0.71 (0.21, 2.36)
Panic disorder	7.7	3.2	4.0	1.92 (0.54, 6.90)	2.37 (0.60, 9.34)	0.81 (0.23, 2.81)	0.97 (0.27, 3.45)
Agoraphobia	6.2	4.9	2.0	3.08 (0.58, 16.32)	3.48 (0.67, 18.13)	2.43 (0.54, 11.04)	2.75 (0.54, 13.93)
Social phobia	15.4	13.0 (n = 184 ^b^)	7.0	2.20 (0.88, 5.48)	1.83 (0.71, 4.75)	1.86 (0.83, 4.17)	1.53 (0.66, 3.57)
Specific phobia	7.7	14.1	11.0	0.70 (0.25, 1.92)	0.71 (0.25, 2.07)	1.28 (0.66, 2.48)	1.68 (0.83, 3.41)
Any anxiety disorder ^d^	24.6	27.0	21.0	1.17 (0.66, 2.07)	1.11 (0.61, 2.02)	1.29 (0.82, 2.01)	1.33 (0.83, 2.13)
Major depression	21.5	13.0 (n = 184 ^b^)	15.0	1.44 (0.74, 2.77)	1.37 (0.69, 2.69)	0.87 (0.48, 1.58)	0.87 (0.46, 1.65)
Suicidal ideation ± attempt	9.2	5.4	3.0	3.08 (0.80, 11.87)	2.10 (0.50, 8.90)	1.80 (0.51, 6.40)	1.45 (0.38, 5.53)
Number of mental health problems ^e^ (past 12 months), mean (SD)	0.74 (1.23)	0.58 (0.98) (n = 183 ^b^)	0.46 (0.93)	1.61 (1.07, 2.41)	1.54 (0.86, 2.73)	1.27 (0.90, 1.80)	1.25 (0.76, 2.03)
**Substance use and offending**							
Substance use, % (past 12 months)							
Daily cigarette smoker	27.7	30.8	21.0	1.32 (0.76, 2.28)	1.07 (0.65, 1.74)	1.47 (0.95, 2.27)	1.13 (0.75, 1.72)
Weekly binge drinking ^f^	13.8	16.2	11.0	1.26 (0.55, 2.87)	1.22 (0.57, 2.58)	1.47 (0.77, 2.81)	1.18 (0.61, 2.29)
Daily cannabis use	16.9	4.3	8.0	2.12 (0.90, 4.98)	1.43 (0.64, 3.22)	0.54 (0.21, 1.40)	0.42 (0.16, 1.08)
Other illicit drug use ^g^	9.2	11.9	11.0	0.84 (0.33, 2.16)	0.66 (0.24, 1.81)	1.08 (0.55, 2.14)	0.92 (0.44, 1.90)
Offending, %							
Property or violent offences since age 18	20.0	23.2	23.0	0.87 (0.48, 1.59)	0.74 (0.40, 1.35)	1.01 (0.65, 1.58)	0.79 (0.51, 1.22)
Number of substance use/offending problems, mean (SD)	0.88 (1.33)	0.86 (1.16)	0.74 (1.14)	1.19 (0.84, 1.67)	0.98 (0.65, 1.49)	1.17 (0.89, 1.54)	0.92 (0.64, 1.33)

VLBW: very low birthweight, RR: relative risk, ARR: adjusted relative risk, CI: confidence interval, and SD: standard deviation. ^a^ Gestational age of VLBW participants stratified into <28 weeks (EPT) and ≥28 weeks (n = 160 born at 28–32 weeks, n = 25 born ≥ 33 weeks) and compared with term controls (≥37 weeks gestation). ^b^ n < 185 for some measures where participants or parent proxies only completed part of the interview. ^c^ Adjusted for sex, ethnicity, breastfeeding, maternal age at childbirth, parental education, childhood family socioeconomic status, and potential selection bias in VLBW cohort. ^d^ Generalised anxiety disorder, panic disorder, agoraphobia, social phobia, and/or specific phobia assessed using standardised diagnostic criteria. ^e^ Suicidality, depression, and/or anxiety disorders listed above. ^f^ Defined as consuming ≥ 6 standard drinks on >50 occasions in the last year. ^g^ Use of any illicit substance other than cannabis and/or abuse of prescription medications for recreation.

## Data Availability

The data presented in this study are available in this article and Supplementary Materials.

## Supplementary Materials

The following supporting information can be downloaded at: https://www.mdpi.com/article/10.3390/jcm13247591/s1, Figure S1: New Zealand VLBW Follow-up Study: Cohort flow chart [49]; Table S1: Demographic and perinatal characteristics of VLBW survivors who were assessed and not-assessed on mental health outcomes; Table S2: 12-month prevalence of mental health disorder or subthreshold mental health symptoms and number of mental health problems including subthreshold disorder over past 12 months in VLBW and controls; Table S3: Mental health, substance use and offending outcomes in VLBW and controls stratified by sex; Table S4: Mental health, substance use and offending outcomes stratified by birthweight; Table S5: Adjusted outcome rate ratios (95%CIs) in VLBW adults compared to term controls, excluding those VLBW with moderate/severe neurosensory disability at age 7–8 years.
