# Supplementary material for: Mental Health Outcomes of a National Cohort of Adults Born with Very Low Birthweight
Source: J Clin Med. 2024 Dec 13;13(24):7591. doi: 10.3390/jcm13247591 (PMC11728432; doi:10.3390/jcm13247591)
Supplement: Supplementary file 1 [file jcm-13-07591-s001.zip › jcm-3349542-supplementary.pdf]

Supplemental Materials

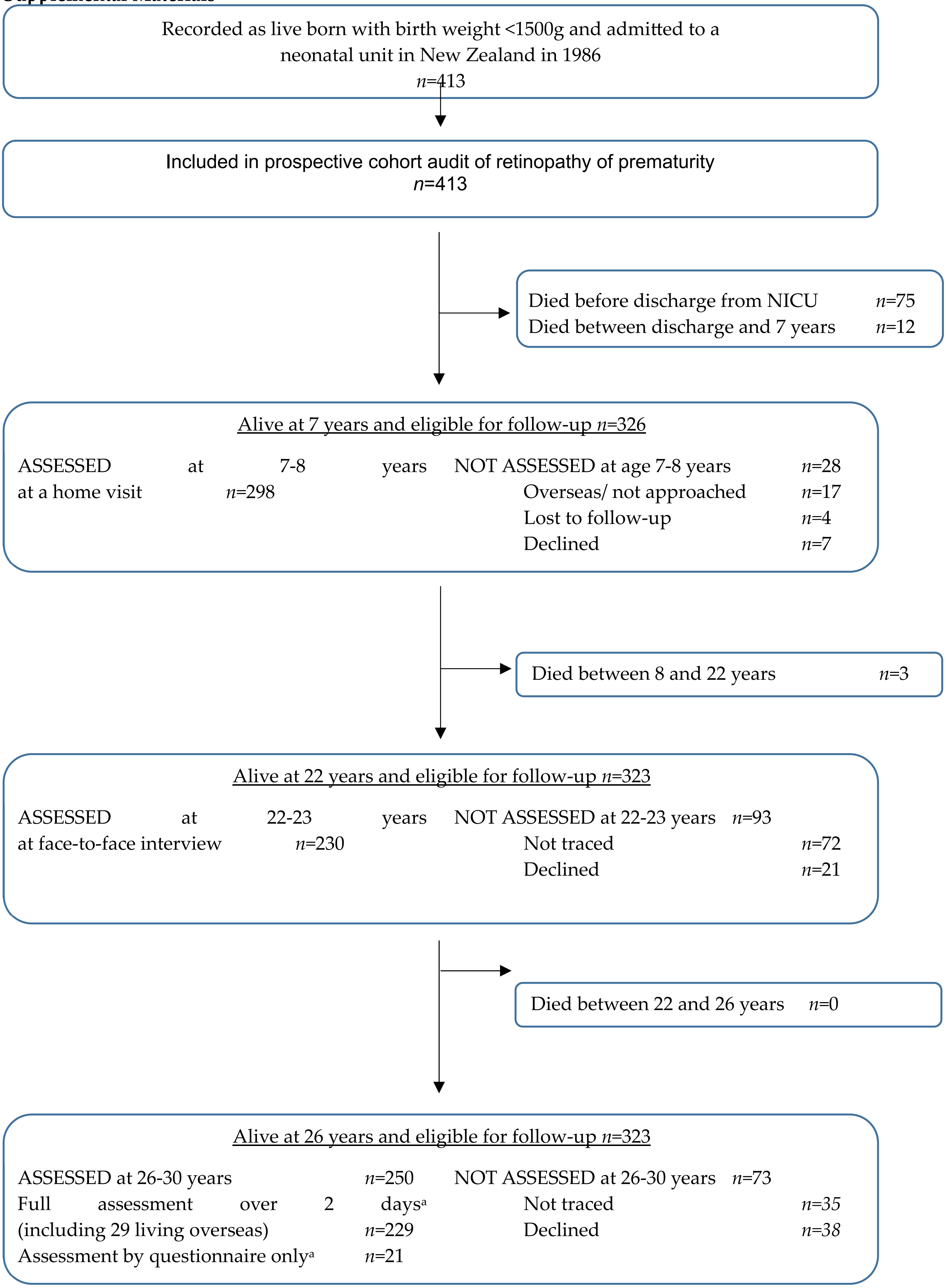

Figure S1. New Zealand 1986 VLBW Adult Follow-up Study: Cohort flow chart. [a]Between February 2013 and November 2016. NICU: Neonatal intensive care unit. 1986 Term-born Control participants: 69 were recruited at 22-23 year assessment; 39 of these, together with an additional 61 new recruits (n=100) were assessed at 26-30 years (Adapted from [49]).

Table S1. Demographic and perinatal characteristics of VLBW survivors who were assessed and not-assessed on mental health outcomes.

Measure	Assessed (n=250)	Not assessed[a] (n=73)	Difference (95%CI)
Male, %	42.8	61.6	-18.8 (-31.6, -6.1)
Māori/Pacific Island ethnicity, %	30.8	35.6	-4.8 (-17.2, 7.6)
Birth weight (g), mean (SD)	1134 (236)	1206 (228)	-72.8 (-134.1, -11.5)
<1000g, %	27.2	21.9	5.3 (-5.7, 16.3)
Gestation (weeks), mean (SD)	29.2 (2.5)	29.5 (2.4)	-0.33 (-0.98, 0.31)
<28 weeks gestation, %	26.0	20.5	5.5 (-5.3, 16.2)
Small for gestational age (<10th centile), %	30.0	24.7	5.3 (-6.1, 16.8)
Antenatal corticosteroids, %	56.4	60.3	-3.9 (-16.7, 8.9)
Respiratory distress syndrome, %	56.4	56.2	0.2 (-12.7, 13.2)
Bronchopulmonary dysplasia[b], %	20.4	23.3	-2.9 (-13.8, 8.0)
Retinopathy of prematurity, %	21.6	12.3	9.3 (0.2,18.4)
Duration breastfeeding (months), mean (SD)	4.5 (5.9)	5.2 (8.3)	-0.67 (-2.56, 1.21)
Parental education[c], mean (SD)	2.1 (0.8)	2.0 (0.9)	0.03 (-0.21, 0.27)
Moderate/severe neurosensory disability[d] (age 7-8 years), %	8.7	11.1	-2.4 (-11.5, 6.7)

VLBW: very low birth weight, SD: standard deviation. [a] Includes 73 survivors with no follow-up (35 not able to be contacted [13 known to be overseas], 38 contacted but declined). [b] Oxygen requirement at 36 weeks post-menstrual age. [c] Parental education scored in 3 levels (no formal qualifications/high school qualifications/tertiary qualifications) based on highest educational attainment of either parent. [d] Moderate or severe disability was defined as: cerebral palsy in non-ambulant children or in ambulant children causing considerable limitation of movement, or bilateral sensorineural deafness requiring hearing aids, or bilateral blindness, or an IQ score of >2 SD below the test mean (<70) on the Revised Wechsler Intelligence Scale for Children.

Table S2. 12-month prevalence of mental health disorder or subthreshold mental health symptoms and number of mental health problems including subthreshold disorder over past 12 months in VLBW and controls.

Measure	VLBW (n=248-250[a])	Controls (n=100)	RR (95% CI)	
			Unadjusted	Adjusted[b]
Generalised anxiety disorder, %	10.1 (n=248)	5.0	2.02 (0.79, 5.12)	1.71 (0.65, 4.49)
Panic disorder, %	9.2	6.0	1.53 (0.64, 3.65)	1.60 (0.65, 3.93)
Agoraphobia, %	7.6	8.0	0.95 (0.43, 2.10)	0.89 (0.38, 2.10)
Social phobia, %	22.9 (n=249)	11.0	2.08 (1.14, 3.80)	1.78 (0.96, 3.30)
Specific phobia, %	30.8	27.0	1.14 (0.79, 1.65)	1.13 (0.76, 1.68)
Any anxiety disorder[c], %	48.0	40.0	1.20 (0.91, 1.58)	1.15 (0.87, 1.52)
Major depression, %	23.7 (n=249)	23.0	1.03 (0.68, 1.57)	1.01 (0.65, 1.56)
Number of mental health problems[d], mean (SD)	1.11 (1.38) (n=248)	0.83 (1.22)	1.34 (1.05, 1.71)	1.26 (0.90, 1.78)

VLBW: very low birth weight, RR: rate ratio, CI: confidence interval, SD: standard deviation. [a] n <250 for some measures where participants or parent proxies only completed part of the interview. [b] Adjusted for sex, ethnicity, breastfeeding, maternal age at childbirth, parental education, childhood family socioeconomic status, and potential selection bias in VLBW. [c] Generalised anxiety disorder, panic disorder, agoraphobia, social phobia and/or specific phobia assessed using standardized diagnostic criteria. [d] Suicidality, depression and/or anxiety disorders listed above.

Table S3. Mental health, substance use and offending outcomes in VLBW and controls stratified by sex.

Measure	Females				Males			
	VLBW (n=143[b])	Controls (n=63)	RR (95% CI)	ARR[a] (95% CI)	VLBW (n=107[b])	Controls (n=37)	RR (95% CI)	ARR[a] (95% CI)
**Mental health**								
12-month prevalence, %								
Generalised anxiety disorder	5.6 (n=142[b])	3.2	1.77 (0.39, 8.12)	1.25 (0.28, 5.56)	3.8 (n=106[b])	5.4	0.70 (0.13, 3.66)	0.35 (0.06, 1.95)
Panic disorder	5.6	6.3	0.89 (0.28, 2.82)	1.01 (0.30, 3.39)	2.8	0.0	-[c]	-[c]

Agoraphobia	8.4	1.6	5.28 (0.70, 39.79)	6.01 (0.81, 44.62)	0.9	2.7	0.35 (0.02, 5.39)	0.07 (0.00, 1.56)
Social Phobia	15.4	6.3	2.42 (0.87, 6.74)	2.03 (0.71, 5.85)	11.3 (n=106[b])	8.1	1.40 (0.42, 4.68)	0.91 (0.25, 3.31)
Specific phobia	16.8	12.7	1.32 (0.63, 2.78)	1.41 (0.67, 2.98)	6.5	8.1	0.81 (0.22, 2.96)	1.38 (0.35, 5.52)
Any anxiety disorder[d]	32.2	23.8	1.35 (0.82, 2.23)	1.33 (0.80, 2.21)	18.7	16.2	1.15 (0.50, 2.65)	1.13 (0.46, 2.77)
Major depression	14.7	19.0	0.77 (0.40, 1.47)	0.78 (0.40, 1.54)	16.0 (n=106[b])	8.1	1.98 (0.61, 6.37)	1.92 (0.54, 6.82)
Suicidal ideation ± attempt	7.7	4.8	1.62 (0.47, 5.59)	1.27 (0.32, 5.04)	4.7	0.0	-[c]	-[c]
Number of mental health problems[e] (past 12 months), mean (SD)	0.75 (1.21)	0.54 (1.20)	1.38 (0.94, 2.04)	1.35 (0.78, 2.33)	0.47 (0.77)	0.32 (0.78)	1.43 (0.76, 2.68)	1.36 (0.55, 3.40)
**Substance use and offending**								
Substance use, % (past 12 months)								
Daily cigarette smoker	25.9	23.8	1.09 (0.65, 1.83)	0.97 (0.59, 1.58)	35.5	16.2	2.19 (1.01, 4.76)	1.50 (0.71, 3.16)
Weekly binge drinking[f]	12.6	9.5	1.32 (0.55, 3.17)	1.24 (0.52, 2.97)	19.6	13.5	1.45 (0.59, 3.58)	1.30 (0.51, 3.31)
Daily cannabis use	4.2	9.5	0.44 (0.15, 1.31)	0.47 (0.15, 1.43)	12.1	5.4	2.25 (0.53, 9.50)	1.01 (0.24, 4.28)
Other illicit drug use[f]	8.4	14.3	0.59 (0.26, 1.32)	0.52 (0.22, 1.21)	15.0	5.4	2.77 (0.67, 11.46)	1.99 (0.46, 8.60)

Offending, %								
Property or violent offences (since age 18)	13.3	19.0	0.70 (0.36, 1.35)	0.62 (0.34, 1.12)	34.6	29.7	1.16 (0.66, 2.04)	0.96 (0.53, 1.76)
Number of substance use/offending problems, mean (SD)	0.64 (1.02)	0.76 (1.33)	0.84 (0.60, 1.20)	0.79 (0.50, 1.24)	1.17 (1.36)	0.70 (0.74)	1.66 (1.09, 2.54)	1.28 (0.81, 2.01)

VLBW: very low birth weight, RR: rate ratio, ARR: adjusted rate ratio, CI: confidence interval, SD: standard deviation. [a]Adjusted for sex, ethnicity, breastfeeding, maternal age at childbirth, parental education, childhood family socioeconomic status and potential selection bias in VLBW. [b]n reduced for some measures where participants or parent proxies only completed part of the interview (indicated in brackets). [c] Effect size not estimable, zero cell. [d] Generalised anxiety disorder, panic disorder, agoraphobia, social phobia and/or specific phobia assessed using standardized diagnostic criteria. [e] Suicidality, depression and anxiety disorders listed above. [f]Defined as consuming ≥6 standard drinks on >50 occasions in the last year. [g]Use of any illicit substance other than cannabis and/or abuse of prescription medications for recreation.

Table S4. Mental health, substance use and offending outcomes stratified by birthweight.

Measure	ELBW (<1000g) (n=68)	VLBW (1000-1499g) (n=180-182[a])	Controls (n=100)	ELBW (<1000g) vs Controls		VLBW (1000-1499g) vs Controls	
				RR (95%CI)	ARR (95%CI)[b]	RR (95%CI)	ARR (95%CI)[b]
**Mental Health**							
12-month prevalence, %							
Generalised anxiety disorder	4.4	5.0 (n=180[a])	4.0	1.10 (0.25, 4.77)	0.83 (0.18, 3.79)	1.25 (0.39, 3.96)	0.83 (0.26, 2.68)
Panic disorder	11.8	1.6	4.0	2.94 (0.92, 9.38)	3.17 (0.97, 10.34)	0.41 (0.09, 1.80)	0.48 (0.11, 2.08)
Agoraphobia	5.9	4.9	2.0	2.94 (0.55, 15.61)	2.97 (0.57, 15.40)	2.47 (0.54, 11.22)	2.98 (0.59, 15.17)

Social phobia	13.2	13.8 (n=181[a])	7.0	1.89 (0.74, 4.83)	1.65 (0.63, 4.29)	1.97 (0.89, 4.40)	1.60 (0.68, 3.74)
Specific phobia	11.8	12.6	11.0	1.07 (0.45, 2.52)	1.08 (0.45, 2.57)	1.15 (0.58, 2.26)	1.51 (0.74, 3.06)
Any anxiety disorder[c]	25.0	26.9	21.0	1.19 (0.68, 2.08)	1.16 (0.65, 2.07)	1.28 (0.82, 2.01)	1.31 (0.82, 2.11)
Major depression	14.7	15.5 (n=181[a])	15.0	0.98 (0.47, 2.05)	0.94 (0.44, 2.03)	1.03 (0.58, 1.84)	1.05 (0.56, 1.95)
Suicidal ideation ± attempt	4.4	7.1	3.0	1.47 (0.31, 7.07)	1.14 (0.21, 6.20)	2.38 (0.69, 8.16)	1.89 (0.49, 7.34)
Number of mental health problems[d] (past 12 months), mean (SD)	0.66 (1.25)	0.61 (0.97) (n=180[a])	0.46 (0.93)	1.44 (0.95, 2.17)	1.38 (0.75, 2.51)	1.33 (0.94, 1.87)	1.31 (0.81, 2.12)
**Substance use and offending**							
Substance use, % (past 12 months)							
Daily cigarette smoker	25.0	31.9	21.0	1.19 (0.68, 2.08)	1.03 (0.62, 1.70)	1.52 (0.98, 2.35)	1.15 (0.76, 1.74)
Weekly binge drinking[e]	13.2	16.5	11.0	1.20 (0.53, 2.75)	1.13 (0.51, 2.47)	1.50 (0.79, 2.86)	1.21 (0.62, 2.36)
Daily cannabis use	10.3	6.6	8.0	1.29 (0.49, 3.38)	0.96 (0.36, 2.55)	0.82 (0.35, 1.95)	0.59 (0.25, 1.39)
Other illicit drug use[f]	13.2	10.4	11.0	1.20 (0.53, 2.75)	1.00 (0.42, 2.36)	0.95 (0.47, 1.91)	0.81 (0.38, 1.70)
Offending, %							

Property or violent offences since age 18	17.6	24.2	23.0	0.77 (0.41, 1.44)	0.67 (0.36, 1.24)	1.05 (0.68, 1.63)	0.81 (0.52, 1.26)
Number of substance use/offending problems, mean (SD)	0.79 (1.30)	0.90 (1.17)	0.74 (1.14)	1.07 (0.76, 1.52)	0.92 (0.57, 1.48)	1.21 (0.92, 1.59)	0.94 (0.65, 1.36)

ELBW: extremely low birthweight (<1000g), VLBW: very low birth weight, CI: confidence interval, SD: standard deviation. [a] n <182 for some measures where participants or parent proxies only completed part of the interview. [b] Adjusted for sex, ethnicity, breastfeeding, maternal age at childbirth, parental education, childhood family socioeconomic status and potential selection bias in VLBW. [c] Generalised anxiety disorder, panic disorder, agoraphobia, social phobia and/or specific phobia assessed using standardized diagnostic criteria. [d] Suicidality, depression and/or anxiety disorders listed above. [e] Defined as consuming ≥6 standard drinks on >50 occasions in the last year. [f] Use of any illicit substance other than cannabis and/or abuse of prescription medications for recreation.

Table S5. Adjusted[a] outcome rate ratios (95%CIs) in VLBW adults compared to term controls, excluding those VLBW with moderate/severe neurosensory disability at age 7-8 years.

**Measure**	**All VLBW**	**Sex specific**		**Gestational Age**		**Birthweight**	
		**Females**	**Males**	**<28 weeks**	**≥28 weeks**	**<1000g**	**1000-1499g**
**Mental Health**							
12 month prevalence, %							
Generalised anxiety disorder	0.80 (0.25, 2.57)	1.17 (0.25, 5.51)	0.39 (0.07, 2.19)	0.92 (0.18, 4.86)	0.77 (0.24, 2.53)	0.92 (0.19, 4.39)	0.77 (0.24, 2.52)
Panic disorder	1.28 (0.40, 4.10)	0.87 (0.26, 2.92)	-[b]	2.06 (0.50, 8.50)	0.97 (0.27, 3.40)	2.92 (0.88, 9.66)	0.48 (0.11, 2.05)
Agoraphobia	2.96 (0.63, 13.96)	5.87 (0.77, 44.91)	0.08 (0.00, 1.36)	2.82 (0.48, 16.68)	3.02 (0.60, 15.19)	3.27 (0.62, 17.30)	2.80 (0.55, 14.38)
Social phobia	1.55 (0.68, 3.52)	1.90 (0.66, 5.48)	1.02 (0.26, 3.95)	1.61 (0.60, 4.31)	1.53 (0.65, 3.59)	1.69 (0.64, 4.45)	1.50 (0.63, 3.54)

Specific phobia	1.28 (0.66, 2.47)	1.34 (0.64, 2.81)	1.26 (0.31, 5.06)	0.45 (0.12, 1.61)	1.66 (0.82, 3.33)	1.06 (0.42, 2.65)	1.38 (0.68, 2.79)
Any anxiety disorder[c]	1.27 (0.81, 1.98)	1.35 (0.81, 2.24)	1.12 (0.45, 2.78)	1.09 (0.59, 2.02)	1.33 (0.84, 2.12)	1.26 (0.70, 2.24)	1.27 (0.79, 2.04)
Major depression	1.02 (0.57, 1.82)	0.74 (0.38, 1.47)	2.12 (0.61, 7.40)	1.28 (0.64, 2.55)	0.93 (0.49, 1.75)	0.85 (0.38, 1.90)	1.09 (0.59, 2.03)
Suicidal ideation ± attempt	1.73 (0.46, 6.55)	1.17 (0.27, 5.05)	- [b]	2.33 (0.55, 9.96)	1.49 (0.37, 5.95)	0.95 (0.15, 6.22)	2.05 (0.50, 8.31)
Number of mental health problems[d] (past 12 months), mean (SD)	1.29 (0.81, 2.06)	1.26 (0.72, 2.19)	1.45 (0.58, 3.60)	1.34 (0.74, 2.42)	1.27 (0.77, 2.08)	1.34 (0.72, 2.50)	1.26 (0.78, 2.06)
**Substance use and offending**							
Substance use, % (past 12 months)							
Daily cigarette smoker	1.21 (0.82, 1.79)	1.00 (0.62,1.62)	1.76 (0.83, 3.76)	1.13 (0.70, 1.85)	1.24 (0.82, 1.86)	1.09 (0.65, 1.80)	1.26 (0.84, 1.89)
Weekly binge drinking[e]	1.29 (0.69, 2.42)	1.29 (0.53, 3.09)	1.45 (0.58, 3.61)	1.34 (0.64, 2.82)	1.28 (0.66, 2.46)	1.28 (0.59, 2.77)	1.30 (0.67, 2.52)
Daily cannabis use	0.77 (0.34, 1.75)	0.52 (0.17, 1.61)	1.21 (0.27, 5.35)	1.85 (0.83, 4.08)	0.23 (0.09, 0.54)	1.19 (0.45, 3.16)	0.63 (0.26, 1.53)
Other illicit drug use[f]	0.94 (0.47, 1.88)	0.56 (0.24, 1.29)	2.29 (0.53, 9.83)	0.73 (0.27, 2.01)	1.00 (0.49, 2.07)	1.14 (0.49, 2.68)	0.88 (0.42, 1.84)
Offending, %							
Property or violent offences since age 18	0.87 (0.58, 1.30)	0.69 (0.38, 1.24)	1.09 (0.61, 1.93)	0.80 (0.44, 1.45)	0.89 (0.58, 1.36)	0.78 (0.44, 1.40)	0.90 (0.59, 1.37)

Number of substance use/offending problems, mean (SD)	1.04 (0.73, 1.47)	0.84 (0.53, 1.31)	1.49 (0.96, 2.30)	1.09 (0.72, 1.63)	1.02 (0.71, 1.47)	1.05 (0.66, 1.67)	1.03 (0.72, 1.49)

VLBW: very low birth weight, CI: confidence interval, SD: standard deviation. [a] Adjusted for sex, ethnicity, breastfeeding, maternal age at childbirth, parental education, childhood family socioeconomic status and potential selection bias in VLBW. [b] Effect size not estimable, zero cell. [c] Generalised anxiety disorder, panic disorder, agoraphobia, social anxiety disorder and/or specific phobia assessed using standardized diagnostic criteria. [d] Suicidality, depression and/or anxiety disorders listed above. [e] Defined as consuming ≥6 standard drinks on >50 occasions in the last year. [f] Use of any illicit substance other than cannabis and/or.
